# Efficient real-world image denoising using multi-scale gaussian pyramids

**DOI:** 10.1038/s41598-025-23942-8

**Published:** 2025-11-17

**Authors:** Asha Rani, Rosepreet Kaur Bhogal

**Affiliations:** School of Electronics and Electrical Engineering, Lovely Professional University, Phagwara, 144411 India

**Keywords:** Image denoising, Gaussian pyramid, Wavelet transform, Real-world images, Multiscale resolution, Image processing, Engineering, Mathematics and computing

## Abstract

The field of image denoising has undergone significant advancements over the years. Recently, Convolutional Neural Networks (CNN) based denoising methods have shown remarkable performance in image denoising. Most of these adopt single-scale features, which may have limitations in denoising real-world images. Real-world noise is complex and non-Gaussian in nature. The multi-scale strategy of the Gaussian pyramid (GP) facilitates the attenuation of noise while preserving image details. Additionally, this multiscale architecture inherently reduces the data’s dimensionality, resulting in decreased computational complexity. Over the past few decades, this method has been employed for image denoising; however, its application to real-world images remains computationally challenging. In this study, we implemented the GP method for denoising X-ray, MRI, non-medical images, and SIDD datasets. Furthermore, its denoising performance is compared with the wavelet transforms (Coiflet4, Haar, Daubechies, and Symlets). Quantitatively, GP achieves a significant improvement in PSNR, SSIM, and computational complexity compared to the wavelet method. PSNR of 36.8024 dB, SSIM of 0.9428, and computational complexity of 0.0046 s have been achieved, thereby offering an effective and practical solution for real-world image applications.

## Introduction

Noise in images is a common problem. It is the unwanted signal that is generally introduced during the acquisition, transmission, and/or reconstruction of an image. Though the noise cannot be altogether eliminated, it can be reduced at acquisition time^1^. Possible ensuing image processing tasks, such as video processing, image analysis, and tracking, are adversely affected; therefore, image denoising plays a crucial role in modern image processing systems.

Image denoising plays a pivotal role in many computer vision and image analysis tasks, including object recognition, medical imaging, remote sensing, and surveillance. The main objective of denoising is to enhance image quality by removing noise while preserving important structural details such as textures, edges, and contours^2^. Noise can arise from various sources, including sensor limitations, transmission errors, or environmental interference during image acquisition. The choice of denoising technique depends on the specific application and the type of noise^3^. The key factors, such as edge preservation, artifact introduction, and computational efficiency, must be carefully considered. Overall, image denoising remains an active area of research, with ongoing efforts to improve performance and adaptability to various noise models^4^.

Real-world noise is complex and unpredictable, which occurs in images captured under practical conditions using imaging devices such as smartphones, digital cameras, or medical scanners^5^. In the medical domain, modalities including X-ray, CT, and MRI are affected by this signal-dependent, non-Gaussian, spatially variant, and more structured^6^. In the past decades, a large number of noise modelling methods have been proposed to remove Additive White Gaussian noise (AWGN) and Mixture of Gaussian (MoG) noise^7^. Despite achieving competitive results, these are not adaptive enough to denoise real-world images^6^. Some existing methods, such as mean filtering, median filtering, and wavelet thresholding, offer simple and computationally efficient solutions^8^. However, these compromise image details^9^. More advanced methods like non-local means^10^, Block-Matching, 3D filtering^11^ and dictionary learning^12^ provide improved performance by exploiting spatial and statistical redundancies, and may face challenges with multilevel real-world noise.

Advancements in deep learning led to more sophisticated approaches^13^. Still, one notable constraint in many existing methods is that they generally focus on deeper and larger Convolutional Neural Networks (CNNs)^14^ where a large number of network parameters are to be learned to represent the noise features. In that case, a trade-off between computational complexity (CC) and denoising quality must be established.

Real-world noise often appears at multiple scales, and effectively addressing it across varying scales remains a significant challenge. Multi-scale approaches have shown competitive performance compared to state-of-the-art denoisers^15^^,^^16^. Each layer captures features or noise components at different scales, which can then be isolated and removed from coarse to fine levels. The Gaussian Pyramid (GP) framework has shown great potential in addressing real-world noise^17^. This framework employs a three-stage process, including noise estimation, denoising, and feature fusion, to effectively handle real-world noise^18^. This decomposition process facilitates noise attenuation at coarser levels while preserving fine details at higher resolutions with improved CC^19^. This denoising method exhibits significant advantages in terms of accuracy, efficiency, and information preservation compared to other multiscale techniques, such as wavelet transforms.

Given the wide range of denoising techniques available in the literature, this study delivers a rigorous comparative evaluation of the GP-based denoising method against wavelet variants, including wavelet methods Coiflet4 (Coif4), Daubechies (db4), Haar, and Symlets (sym4), across diverse X-ray, MRI, Non-medical, and SIDD datasets. It provides a fine-grained analysis of filter selection and justifies the 5-layer architecture, while quantifying performance using PSNR, SSIM, MSE, RMSE, VIF, FOM, MAE, computational efficiency, and standard deviation (SD), along with statistical tests, including paired t-test, and Wilcoxon Signed-Rank Test. The findings offer practical guidance for real-world deployment, highlighting scenarios where GP achieves an optimal balance between structural fidelity and processing cost. Most existing image denoising studies are evaluated on the SIDD and other natural image benchmarks. In addition to these datasets, our work also focuses on medical image datasets, where preserving fine details is critical for accurate diagnosis.

### Objective of the paper

The objective of this research is to:

- Implement a GP-based approach for denoising real-world images.
- Evaluate the performance of this approach against wavelet transforms (coif4, db4, Haar, sym4).
- Demonstrate the potential of GP in both preserving structural details and reducing noise.

### Literature survey

In recent years, a large amount of work has been proposed on image denoising. Fixed noise removal from images has been well-studied; however, limited work has been done on real-world image denoising. Real-world noise is variant and random in nature, which may not be identified efficiently by using a single noise level. Therefore, image denoising remains a significant challenge in real-world images.

## Noises in real-world images

**Fig. 1:**
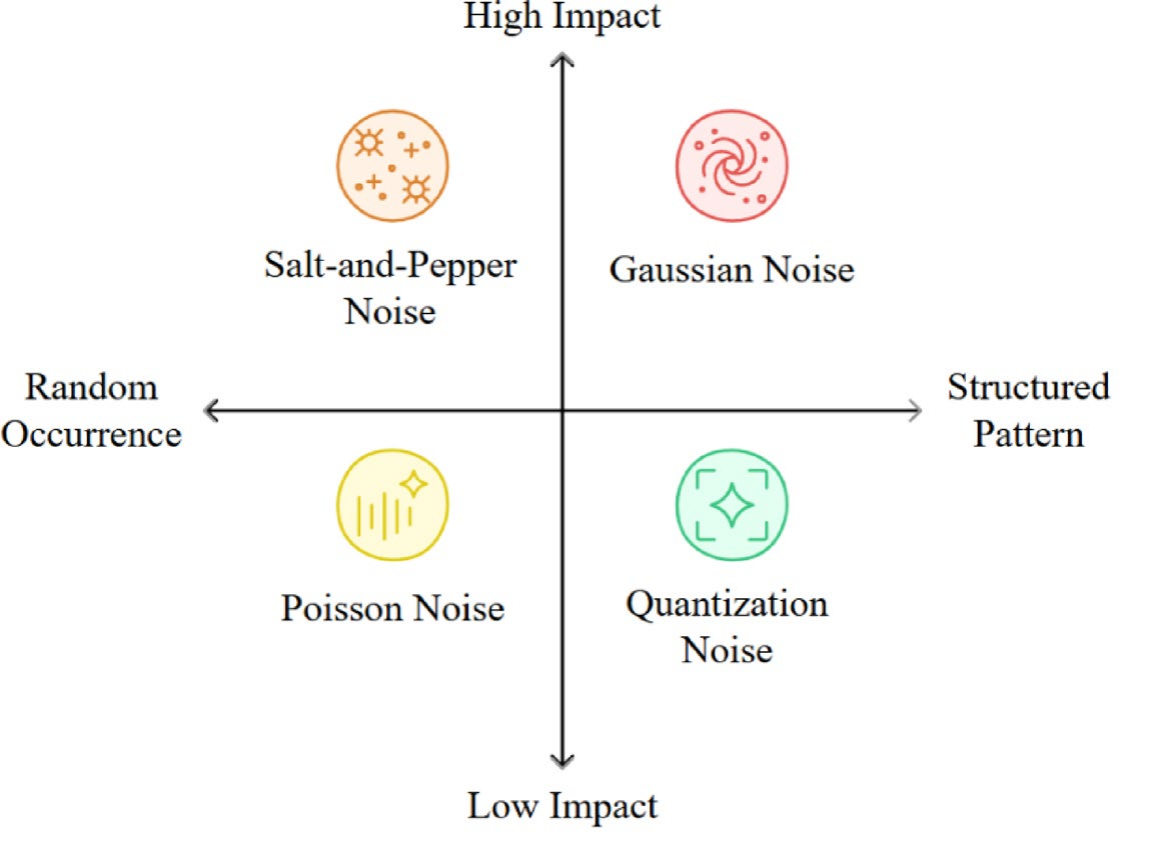
Types of image noise and their characteristics, illustrating their effects on image quality.

**Fig. 2:**
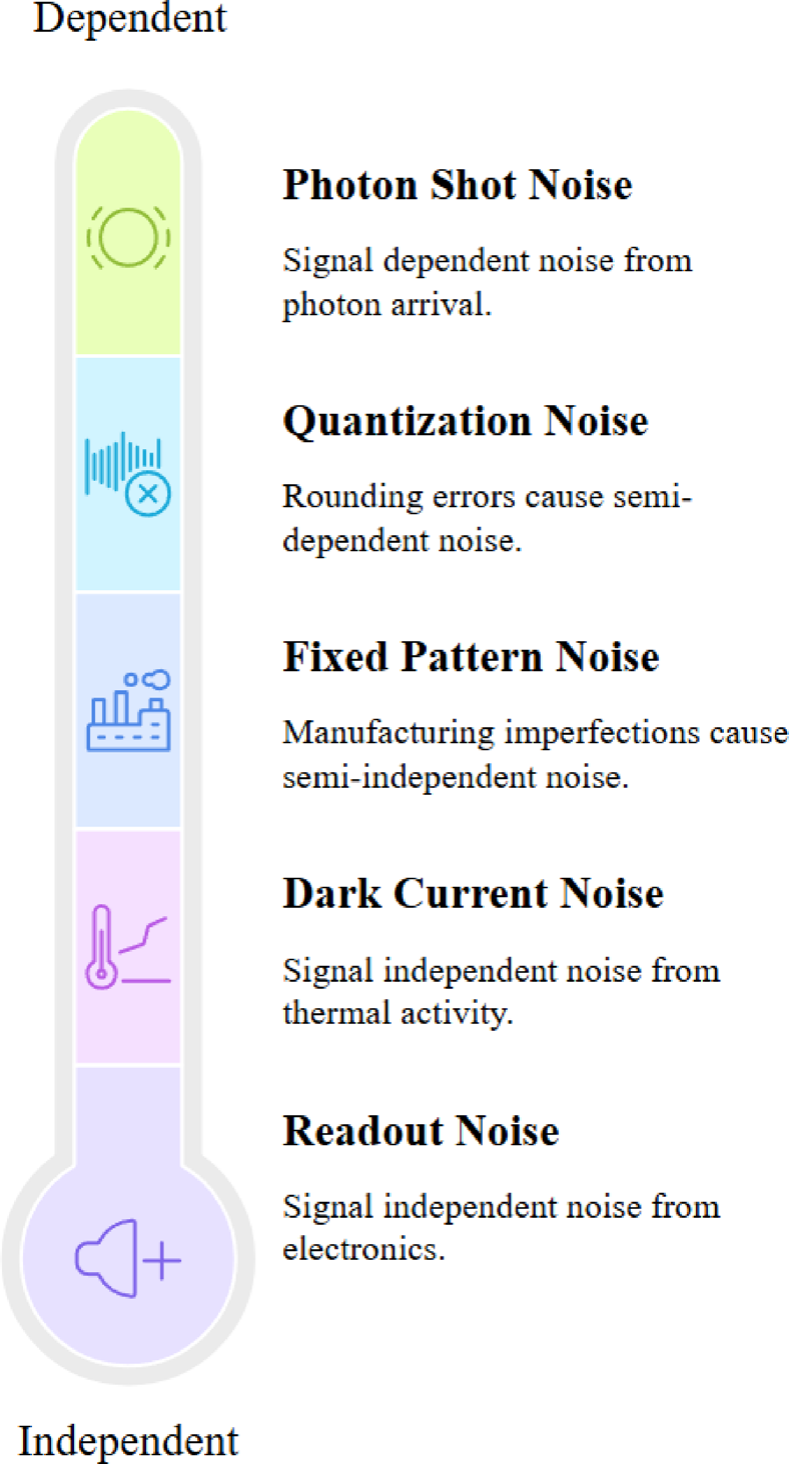
Categorization of real-world image noise based on signal dependency levels.

Images taken in real-world situations often encounter various types of noise, including Gaussian, Salt and Pepper, quantization, and Poisson noise. Each type affects the image differently^20^. Figure 1 illustrates that salt and pepper noise has a notable effect and occurs randomly, likely due to sensor issues or transmission errors. Gaussian noise also has a significant impact, creating a statistical distribution due to sensor thermal noise and electronic circuit fluctuations. Poisson noise and quantization noise tend to have less impact and originate during the image generation and digitization. Figure 2 illustrates real-world image noise types based on the signal dependency levels, which stem from physical processes. Photon shot noise is signal-dependent when dark current and read-out noise are signal-independent. Fixed pattern noise arises across the sensor array because of non-uniformity in pixel response. This variability of noise profiles challenges the assumption of fixed noise in denoising models and emphasizes the necessity for denoising methods that account for signal dependency across the entire dynamic range.

## Image denoising methods

Image denoising is a fundamental process in computer vision for enhancing image quality by reducing noise. In general, mathematically, the image denoising problem can be modelled as

1 $$y = x + n$$

where y is the noisy image, x is the unknown clean image, and n represents the noise. This noise can be estimated by various methods. The purpose of the denoising method is to decrease the noise without compromising the important details of the image. These can be categorized as classical and advanced methods.

### Classical image denoising methods

Various methods have been proposed for image denoising, such as spatial, transform, and statistical domain filtering. Most of these methods are based on linear or statistical models, which may limit their ability to capture the complexities of real-world images. Table 1 presents the image denoising methods based on their domain, noise handling capability, edge preservation, and CC. While mean and median filters^21^ have good computational efficiency, yet offer limited performance in retaining important image details. Transform domain filters, such as the Discrete Cosine Transform (DCT)^22^ and Wavelet Transform (WT)^23^, provide enhanced performance for high-quality and multi-level noise while maintaining moderate edge preservation. Statistical methods, including the Wiener filter^22^ and Non-Local Means (NLM)^24^, demonstrate strong adaptability to various noise types while maintaining excellent edge retention, albeit at the cost of increased computational complexity. We must consider trade-offs among noise reduction, edge preservation, and computational demands.

**Table 1:** Classical image denoising methods.

**Characteristic**	**Mean filter**^21^	**Median filter**^21^	**Gaussian filter**^7^	**DCT**^22^	**Wavelet transform**^23^	**Wiener filter**^22^	**NLM**^25^
Domain	Spatial	Spatial	Spatial	Transform	Transform	Statistical	Statistical
Noise type	Gaussian	Salt-and- pepper	Gaussian	High-frequency	Various scales	Gaussian	Various
Edge Preservation	Poor	Good	Moderate	Good	Good	Moderate	Excellent
Complexity	Low	Low	Low	Moderate	Moderate	High	High

### Advanced image denoising techniques

With the enhancement in deep learning (DL), there has been a shift toward data-driven image denoising methods, such as CNN and other machine learning-based methods. These denoising approaches employ cutting-edge computational models to reduce noise and preserve key structural details. Multiple advanced image denoising methods include model-based methods, DL approaches, variational and generative models, hybrid and multiscale strategies.

- Model-based approach involves optimization concepts (such as total variation method and sparse coding) using noise priors^26^. They are versatile and interpretable, but require human intervention, which limits their scalability. They often generate noise artifacts, impeding their generalization to real-world scenarios.
- Learning-based denoising approaches leverage CNNs to model noise distribution. They are effective; however, they require large-scale datasets and often lack interpretability. While these approaches are good at preserving image details, they tend to affect interpretability. So, it becomes challenging to debug or optimize them for better performance in specific applications^27^. Technologii et al. proposed a denoiser using CNNs, which has shown notable enhancements over classical techniques, albeit at the cost of CC^28^.
- Generative models such as Generative Adversarial Networks (GANs)^29^ and variational autoencoders (VAEs)^30^ generate clean images by learning the image distribution.
- Multiscale Techniques: Multiscale representations have emerged as key in the image denoising task because of their ability to capture image structures at different resolutions. While WT has been extensively used for this purpose, GP provides a simpler yet powerful alternative, which involves iterative low-pass filtering and down-sampling, allowing efficient noise suppression across scales while preserving structural information^27^.

## Literature review

Over the course of several decades, extensive research has been dedicated to developing robust and well-structured techniques for image denoising. Many of these targeted fixed noise patterns, typically modeled as Gaussian noise^31^. However, these methods are not flexible and adaptive enough to address the complex and spatially varying characteristics of real-world noise^2^. To address this, Xu et al^32^ proposed prior learning approach that requires human interference, whereas Majed et al.^11^ proposed a blind denoising technique to remove fixed Gaussian noise. Wavelet-based methods depend on the proper selection of a threshold and assumptions about noise for effective denoising^33^. Some studies have proposed enhanced threshold functions to address denoising, leading to an increase in computational cost^34^. Several model-driven methods have been explored. Buades et al. proposed a non-local means filter, which leverages the presence of self-similarity of features in the image for denoising^25^. Xu et al. further introduced a patch grouping-based algorithm to reduce redundancy between similar patches^25,35^. However, it shows limitations for spatially variant noise, which often occurs in real-world images. Xiao et al. extended NLM to a multiscale framework for denoising^36^. Panigrahi et al. proposed an avenue for multiscale NLM by combining curvelet domain processing with NLM filtering to minimize artifacts, achieving a PSNR of 30.526 and an SSIM of 0.896 with a noise density of 30^37^. With the advent of deep learning, CNN-based approaches have gained prominence in image denoising due to their ability to learn complex noise patterns and achieve high PSNR^38^. Model-based^26^ and learning-based techniques using a pattern learning approach, such as DnCNN^38^, Noise2Void^39^, RIDNet^40^, and autoencoders^41^ have shown significant efficacy. Another neural network-based approach employed a dual-attention mechanism, achieving promising results^42^. Hybrid methods combining GP, CNN, and DNN have been developed^43–47^ which offered enhanced performance for image denoising while incurring computational complexity. To further improve denoising, Zhang et al.^48^ provide a framework integrating GP decomposition with a conditional GAN. Additionally, multilevel frameworks are developed by Lam et al.^49^, Ma et al.^50^, Zhong et al.^51^, and others^11,52^. Asem Khmag also proposes a fast and accurate denoising method that integrates pulse-coupled neural networks, wavelet filtering, and regularization of the Perona–Malik equation, achieving improvements in PSNR and SSIM of 0.85–1.54 dB and 0.0132–0.1521, respectively^53^. All these methods require aggressive training, which may limit their computational efficiency.

**Fig. 3:**
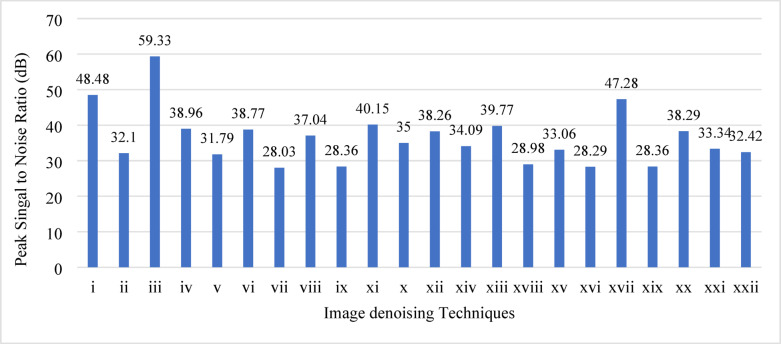
PSNR performance of different image denoising methods.

Table 2 lists the image denoising methods, along with their corresponding PSNR values and references. Figure 3 illustrates the performance of all these image denoising methods. This bar chart displays the denoising results of all these methods achieved on both synthetic and real-world image datasets. As shown in Fig. 3 and Table 2, GP presents a promising approach for image denoising. It achieved better results with PSNR values of 48.48^54^ and 39.77 dB^50^. A hybrid method combining residual learning image denoising (RLID), direct image denoising (DID), GAN, and CNN achieved the highest PSNR of 59.33 dB^19^ when classical and standalone methods achieved 31 and 32 dB^22^,^13^. In another GAN-based denoising method integrated with semi-soft thresholding, an improvement of 2.24 dB in PSNR has been achieved relative to the state-of-the-art studies with the BSE68 and Waterloo exploration datasets^55^.

**Table 2:** Reported PSNR values of different image denoising methods from existing studies with references.

Reference labels	Image denoising methodReferences	PSNR (dB)
i	Zhao et al.^54^	48.48
ii	Bian et al.^13^	32.1
iii	Khan et al.^19^	59.33
iv	R. Ma, Hu, et al., 2020^48^	38.96
v	Lee & Ding^22^	31.79
vi	R. Ma, Hu, et al.^50^	38.77
vii	R. Ma et al.^42^	28.03
viii	T. Wang et al.^18^	38.06
ix	Zheng et al.^43^	38.26
x	Sundarrajan et al.^46^	35
xi	Song et al.^47^	40.15
xii	S. Zhang & Lam^49^	28.36
xiii	R. Ma, Hu, et al., 2020 ^50^	39.77
xiv	D. Zhang et al.^56^	34.03
xv	Z. Wang et al.^52^	33.06
xvi	El Helou & Susstrunk^11^	28.29
xvii	L. Li et al.^57^	47.28
xviii	Feng et al.^36^	28.98
xix	S. Zhang & Lam^49^	28.36
xx	Chihaoui & Favaro^58^	38.29
xxi	Yin et al.^59^	33.34
xxii	Zhang et al.^60^	32.42

**Fig. 4:**
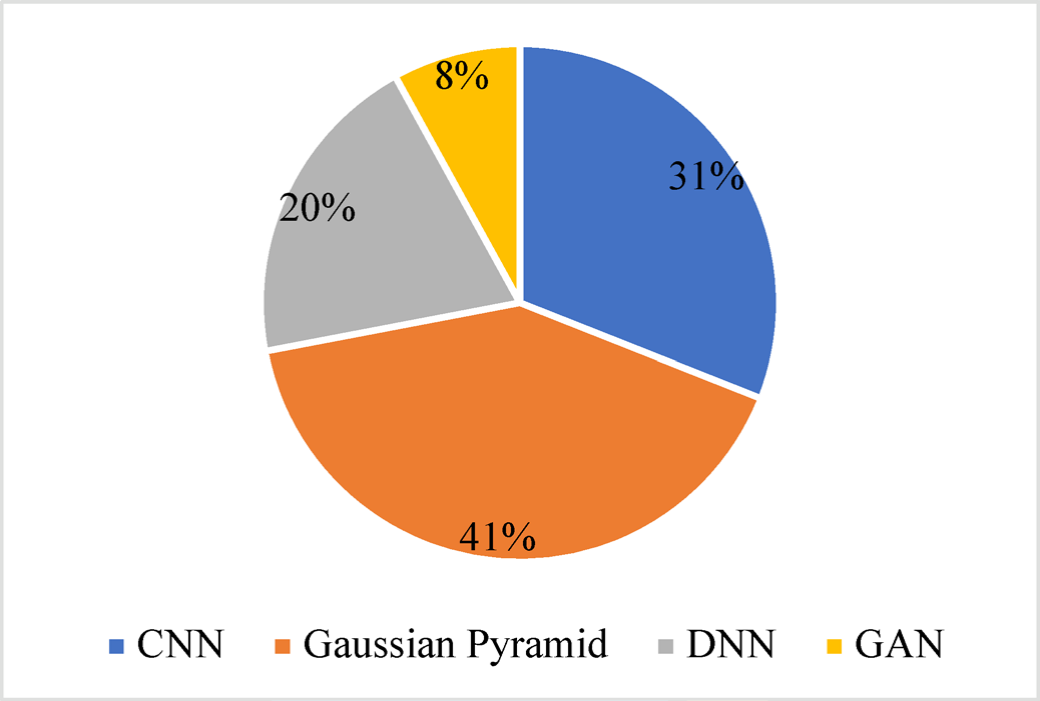
Overview of Methodological trends in multiscale image denoising.

To understand the prevailing methodological trends in multiscale image denoising, a comprehensive review of the publications is conducted. Figure 4 presents the frequency of studies using multi-scale techniques, including CNN, GP, Deep Neural Networks (DNN), and GAN, employed in the literature on image denoising. This distribution suggests a strong research interest in CNNs and GPs due to their effectiveness in capturing spatial hierarchies and reducing noise across different scales.

**Fig. 5:**
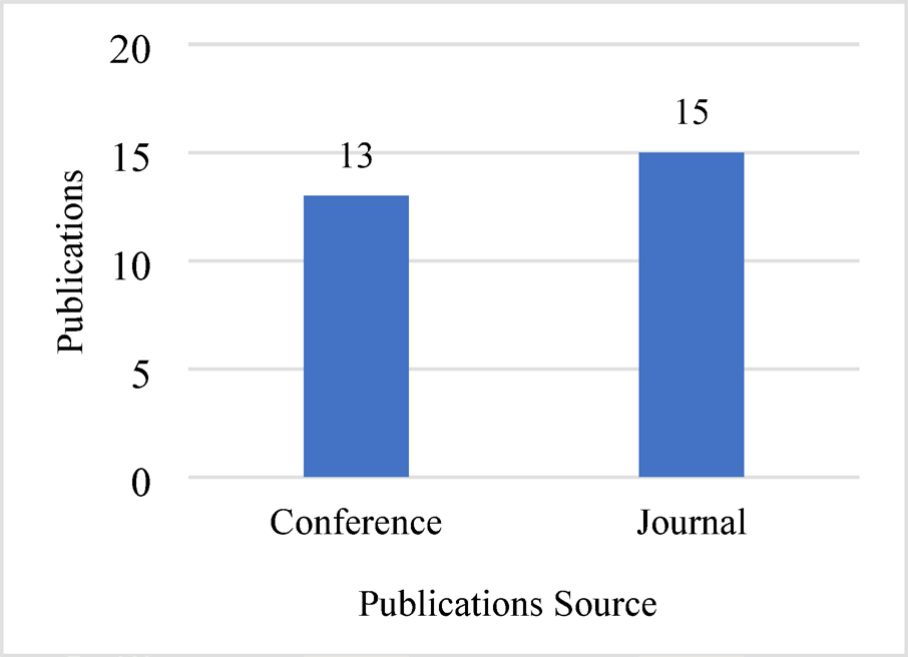
Source-wise distribution of research papers on Gaussian Pyramid (GP) based image denoising.

To assess the dissemination pattern of research on the GP method, a source-wise analysis of published papers was done. Figure 5 (bar chart) presents the source-wise distribution of GP-based research publications across major publication sources. Out of 28 papers, 13 are conference-based and 17 are journal-based. Table 3 presents literature spanning from 2013 to 2024, covering a diverse set of denoising techniques, including CNNs, DNNs, GANs, GP, and hybrid methods. GP-based approaches have been consistently used since 2018, with a notable increase in 2024, in addition to hybrid methods that combine CNN and autoencoders. Recent trends show an increasing interest in multi-model and hierarchical strategies for enhancing denoising performance.

**Table 3:** Literature span of Image denoising techniques.

Year of publication /Methods	Wavelet Transform	GAN	GP, CNN and autoencoder	Gaussian Filter	DnCNN	GaussianPyramid	CNN	DNN
2024			✓			✓	✓	
2023	✓	✓		✓	✓		✓	
2022							✓	
2021		✓					✓	✓
2020					✓	✓	✓	✓
2019		✓					✓	✓
2018						✓	✓	
2017	✓						✓	
2015							✓	✓
2013							✓	

While GP has been previously employed in denoisers, they have predominantly focused on synthetic Gaussian noise or domain-specific applications. They often lack the adaptivity required for diverse and complex real-world noise and in maintaining image structures, necessitating the integration with advanced techniques such as neural networks to overcome these limitations^48^. Some are assuming a fixed Gaussian noise model. Zhang & Lam^49^ and Zhao, et al^54^ have used a multi-level structure and exhibit good results. However, it focuses on synthetic datasets, such as Kodak, BSD65, and SIDD, with simulated noise, which limits its generalisability to practical scenarios. Chihaoui et al. proposed a Mask, Impaint, and Denoise (MID) framework using unsupervised learning. Masking and impainting process involving multiple rounds of training and iterations, which is likely to increase the computational complexity^58^.

GP methods, though computationally efficient for multiscale representation, often induce blurring and loss of fine structures. This may limit their effectiveness in detail-sensitive tasks^19^. These are often inadequate for variable and complex noise arising from sensor imperfections in denoisers^61^ and often face challenges in non-uniform noise distribution conditions, like underwater imaging^62^ Henceforth, hybrid approaches integrating DL and GP are recommended for achieving robust image denoising.

Existing literature mainly favors wavelet or deep learning methods, leaving a gap in the systematic evaluation of GP-based techniques. Moreover, current studies using this multiscale approach often focus on specific modalities. Additionally, comparative research on GP-based denoising across multiple imaging modalities, including MRI, X-ray, Non-medical images, and the SIDD datasets, remains limited, as most studies concentrate on a single modality. This study aims to address this gap by implementing a GP-based denoising framework and benchmarking its performance against the wavelet method using real-world image datasets.

## Methodology

To assess the effectiveness of multiscale denoising strategies, this work implements a GP method and compares its performance with WT techniques, specifically Haar, Daubechies, Coiflet-4, and Symlets transforms. While both methods aim to enhance image quality by isolating noise components, they employ distinct mathematical models for image decomposition. Noise is commonly characterized by a mean value of zero and a variance σ^2^, and it can be mathematically represented as:

2 $$I_{n} \left( {x,y} \right) = I\left( {x,y} \right) + N\left( {x,y} \right)$$

where I(x,y) is the clean image and N(x,y) represents the noise, which may follow a Gaussian or real-world distribution depending upon the imaging conditions. I_n_(x,y) is the noisy version of the image^8^, and (x,y) are pixel coordinates in the image. The aim of denoising is to estimate the original clean image I from the noisy observation I_n._ Denoising methods can be parametric or non-parametric. Classical denoising methods are fast, interpretable, and effective for simple or known noise, while struggling with complex or real-world noise. Learning-based methods achieve state-of-the-art performance and can generalize to complex noise patterns; however, they are limited in computational efficiency and interpretability. The motivation behind this study is to evaluate the effectiveness of multi-resolution strategies rooted in spatial-domain smoothing versus frequency-domain decomposition for noise suppression. The detailed explanations of these multiscale denoising approaches are as follows.

### Gaussian pyramid

A GP is a hierarchical and multi-resolution representation of an image. The process involves repeatedly applying Gaussian smoothing to generate images at pro smaller scales. Hence, it is valuable for real-world image denoising, where multiple levels help in separating noise from image content.

#### Gaussian pyramid construction

**Fig. 6:**
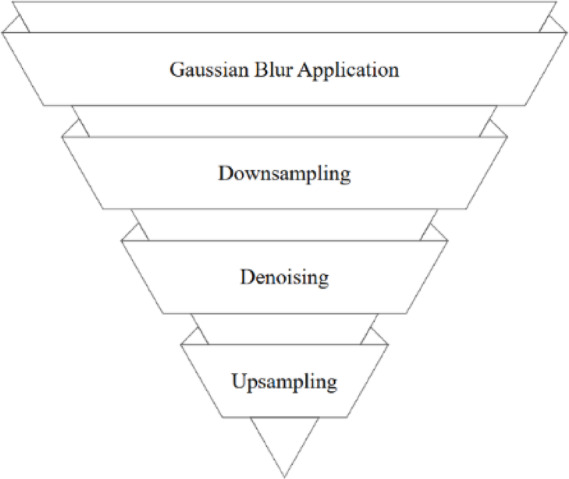
Schematic representation of Gaussian Pyramid construction for multiscale image analysis.

Consists of progressively down-sampled versions of an image, where each level is obtained by convolving the original image with a Gaussian kernel Gσ(x,y), to reduce high-frequency components and suppress noise^63^. The kernel is defined by:

3 $$G_{\sigma } \left( {x,y} \right) = \frac{1}{{2\pi \dot{\sigma }^{2} }}e^{{ - \frac{{x^{{2 + y^{2} }} }}{{2\sigma^{2} }}}}$$

where σ is the standard deviation of the Gaussian kernel, which controls the level of blurring, and (x,y) are the spatial coordinates of the kernel centered around zero. These define the position of a pixel relative to the center of the Gaussian filter kernel. Figure 6 illustrates that after blurring, the image is downsampled to create its reduced-resolution version. The smoothing and down-sampling steps are repeated iteratively until the desired number of pyramid levels is achieved.

Mathematically, this downsampled image at level l is computed as:

4 $$I_{l + 1} \left( {x,y} \right) = \mathop \sum \limits_{i = - k}^{k} \mathop \sum \limits_{j = - k}^{k} G\left( {i,j} \right)I_{l} \left( {2x + i,2y + j} \right)$$

where I_l_(x,y) is the image at level l, G(i,j) is the Gaussian filter kernel, and (x,y) are pixel coordinates in the image.

#### Multiscale denoising process

At each level of the pyramid, a denoising technique is applied to reduce noise. At the finest level (Level 0), a bilateral filter is utilized to attenuate high-frequency noise while maintaining edge sharpness and fine texture details with a neighborhood parameter of 5 and an intensity parameter of 7. This level corresponds to the original resolution of the image, ensuring that high-frequency details such as edges and boundaries remain intact during denoising. Intermediate levels (levels 1 and 2) are processed using median filtering (MF) with a kernel size of 3X3, which is effective in mitigating localized and impulsive noise. Downsampling in the GP compresses the image, causing scattered noise to cluster and become more detectable. Applying MF at lower resolutions avoids blurring delicate textures and edges in the original image. At the coarsest levels (Levels 3 and 4), Gaussian filtering (GF) with a standard deviation of 0.7 and kernel size of 5X5 is applied. These levels represent low-frequency and spatially homogeneous components, and GF here smooths broad intensity variations without affecting finer details, thereby contributing to a smoother and more coherent reconstruction. In GP, five levels are preferred as these many levels capture a broad range of noise, from fine textures (top layers) to coarse structures (bottom layers), enabling effective noise separation across scales. Layers less than this may not capture the full scale of noises present in real-world images, and deeper pyramids (with more than 5 layers) increase memory usage and processing time, incurring computational cost.

#### Image reconstruction

Following the application of denoising filters at each level of the GP, the reconstruction process commences from the coarsest level and progresses to the finest level. Once denoising is completed at each level, the denoised image is upsampled and combined with the denoised image from the next finer level, preserving the fine details. Using multiple levels ensures that fine details are maintained while reducing unwanted noise.

The mathematical expression for up-sampling and interpolation is:

5 $$I_{l + 1} \left( {x,y} \right) = \mathop \sum \limits_{i = - k}^{k} \mathop \sum \limits_{j = - k}^{k} G\left( {i,j} \right)I_{l} \left( {\frac{x}{2} + i,\frac{y}{2} + j} \right)$$

where missing pixels are estimated using interpolation techniques.

### Wavelet transform

Wavelet methods employ orthogonal basis functions to decompose the image into approximation and detail coefficients. The core principle involves the decomposition of an image into multiple sub-bands using discrete WT, thereby isolating different frequency components at different levels.

**Fig. 7:**
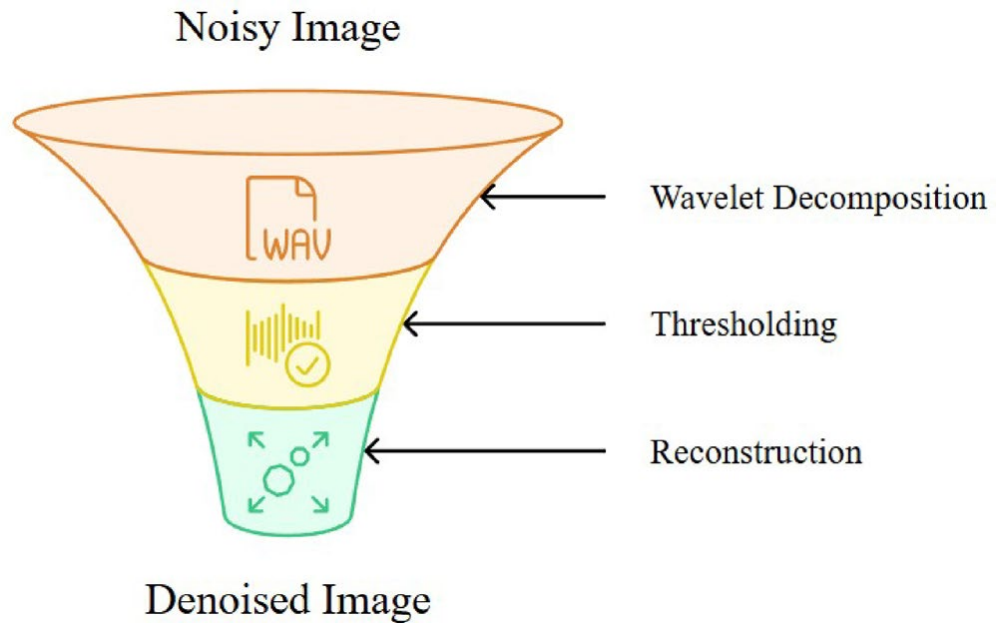
Overview of the Wavelet transform for multiscale image denoising.

Figure 7 illustrates the denoising process by using the wavelet transform method, comprising all three primary stages. The noisy image is first decomposed into approximation and detail coefficients through multi-level wavelet decomposition. Subsequently, noise is suppressed by applying soft or hard thresholding to the detail coefficients. Finally, the image is reconstructed using the inverse WT.

#### Wavelet decomposition

Noisy image is decomposed into four sub-bands as follows

6 $$W\left( {I_{n} } \right) = \left\{ {LL, LH, HL, HH} \right\}$$

where LL is the approximation. LH, HL, and HH are the vertical, horizontal, and diagonal details, respectively.

Wavelet functions can be represented as

7 $$W_{j,k} = \mathop \sum \limits_{x,y} I_{n} \left( {x,y} \right)\psi_{j,k} \left( {x,y} \right)$$

8 $$V_{j,k} = \mathop \sum \limits_{x,y} I_{n} \left( {x,y} \right)\Phi_{j,k} \left( {x,y} \right)$$

with ψ being the wavelet 0unction and Φ being the scaling function.

#### Thresholding

The noise is suppressed by applying soft or hard thresholding to the detail coefficients.

#### Hard thresholding

9 $$\hat{W}_{j,k} = \left\{ {\begin{array}{*{20}c} {W_{j,k} ,} & {\left| {W_{j,k} } \right| \ge T} \\ 0 & {\left| {W_{j,k} } \right| \ge T} \\ \end{array} } \right.$$

Coefficients are kept unchanged if the coefficient is equal to or greater than the threshold value; otherwise, it is set to zero (assumed to be zero).

#### Soft thresholding

10 $$\hat{W}_{j,k} = sign\left( {W_{j,k} } \right).\max \left( {\left| {W_{j,k} } \right| - T,0} \right)$$

It shrinks the magnitude of large coefficients by T and discards small ones.

#### Reconstruction

Finally, the image is reconstructed by applying the inverse Wavelet Transform (IWT) to the threshold coefficients

11 $$\hat{I}\left( {x,y} \right) = IWT\left( {\left\{ {LL, \hat{L}H \hat{H}L, \hat{H}H} \right\}} \right)$$

In this study, we employed orthogonal wavelet families, Haar, Daubechies, Coiflet-4, and Symlets to analyze the denoising performance at various scales.

Previous work has demonstrated that GP is effective in applications such as texture analysis, image compression, and multi-scale denoising. However, its application in real-world image denoising remains relatively underexplored, particularly in the presence of complex noise patterns found in real images.

### Evaluation metrics

The quality of an image is determined by both objective and subjective evaluation. For subjective evaluation, the image has to be observed by a human expert. The human visual system is highly complicated; therefore, objective evaluation is preferred to measure the image quality. Various matrices are available for the objective evaluation of an image denoising method. Some of these are mean squared error (MSE), peak signal-to-noise ratio (PSNR), and structural similarity index measure (SSIM). CC also plays an important role in evaluating the system’s performance.

#### Mean squared error (MSE)

Mean square error is the average squared difference between the estimated values and the actual values. It is given by

12 $$MSE = \frac{1}{mn}\mathop \sum \limits_{i = 0}^{m - 1} \mathop \sum \limits_{j = 0}^{n - 1} \left[ {I\left( {i,j} \right) - I_{n} \left( {i,j} \right)} \right]^{2}$$

where I(i,j) is the noise-free image and I_n_(i,j) is the noisy image of size m x n.

#### Root mean squared error (RMSE)

RMSE is a measure of the average error between the estimated and actual values. It is directly related to MSE.

13 $$RMSE = \sqrt {MSE}$$

#### Mean absolute error (MAE)

MAE evaluates the pixel-wise average absolute difference between the reference and denoised image and is less sensitive to large outliers.

14 $$MAE = \frac{1}{MN}\mathop \sum \limits_{i = 1}^{M} \mathop \sum \limits_{j = 1}^{N} \left| {I\left( {i,j} \right) - K\left( {i.j} \right)} \right|$$

where I(i,j) is the pixel value of the reference image, K(i,j) is the pixel value of the denoised image with size MXN.

#### Peak signal-to-noise ratio (PSNR)

PSNR is the ratio between the maximum possible power of a signal and the power of corrupting noise that affects the fidelity of its representation^64^. Because many signals have a very wide dynamic range, PSNR is generally represented as a logarithmic quantity using the decibel scale. This is linked to the commonly known mean squared error as follows:

15 $$PSNR = 10\;log_{10} \left( {\frac{{MAX_{i}^{2} }}{MSE}} \right)$$

where MAX_i_ is the maximum possible pixel value of the image. When the pixels are represented using 8 bits per sample, the maximum value is 255. So, PSNR can also be represented as

16 $$PSNR = 10\;log_{10} \left( {\frac{{255^{2} }}{MSE}} \right)$$

#### Structural similarity index measure (SSIM)

SSIM is a method for predicting the perceived quality of images^64^. SSIM is used for measuring the similarity between two images. It calculates changes in the luminance, contrast, and structure difference between them. The difference with other techniques, such as MSE or PSNR, is that these approaches estimate absolute errors. Structural information refers to the concept that pixels exhibit strong interdependencies, particularly when they are spatially close. These dependencies carry important information about the structure of the objects in the visual scene. The SSIM index is calculated on various windows of an image^8^.

The measure between two windows x and y of common size NXN is represented as follows

17 $$SSIM\left( {x,y} \right) = \frac{{\left( {2\mu_{x} \mu_{y} + c_{1} } \right)\left( {2\sigma_{xy} + c_{2} } \right)}}{{\left( {\mu_{x}^{2} + \mu_{y}^{2} + c_{1} } \right)\left( {\sigma_{x}^{2} \sigma_{y}^{2} + c_{2} } \right)}}$$

where σ_x_ and σ_y_ are the variances of x and y, respectively, and σ_xy_ is the covariance of x and y. μ_x_ and μ_y_ are the averages of x and y. c_1_ = (k_1_L)^2^ and c_2_ = (k_1_L)^2^ are two variables to stabilise the division with a weak denominator. L is the dynamic range of the pixel value, and k1 and k_2_ are 0.01 and 0.03, respectively.

#### Visual information fidelity (VIF)

VIF is a full-reference quality metric that evaluates the visual information retained in a noised image with reference to the original image. is preserved in noise. It is based on mutual information theory and varies from 0 (no useful information) to 1 (perfect fidelity). It correlates with visual perception than PSNR.

#### Figure of merit (FOM).

FOM is often used to evaluate how well the edges are retained after processing the image. It varies from 0 (poor edge match) to 1 (perfect edge preservation).

#### Computational complexity (CC)

CC refers to the time and memory resources required to process an image, remove noise, and preserve important details. It is a key factor, especially for real-world applications. It affects runtime speed, memory usage, and energy consumption. Different denoising algorithms vary in complexity based on their mathematical operations, the image size, and the type of noise. GP and WT are both multi-resolution approaches for image denoising, but they differ in CC, efficiency, and effectiveness.

#### Standard deviation (SD)

Standard deviation is the statistical measure that indicates the dispersion of a dataset from its mean value. It provides insight into the algorithm stability and residual error characteristics. Performance metrics such as PSNR or SSIM are reported as mean with SD values. SD indicates the consistency across the dataset, and the mean value represents the average effectiveness.

Mathematically, it is represented as

18 $$\sigma = \sqrt{\frac{1}{N}} \mathop \sum \limits_{i = 1}^{N} (x_{i} - \mu )^{2}$$

where σ is the standard deviation, x_i_ is each individual parametric value, N is the number of samples, and µ is the mean value.

#### Paired t-test

This statistical testing is performed to evaluate the effectiveness of denoising results on the same set of images. Paired t-tests are performed on PSNR values obtained from two different methods as follows:

19 $$t = \frac{{\sum \left( {x_{1} - x_{2} } \right)}}{{\frac{s}{\sqrt n }}}$$

where x_1_ and x_2_ are the difference means of the pairs, s is the standard deviation, and n is the sample size, which is 10 in this study. A high t-value and a low *p*-value imply that method A significantly outperforms method B.

### Experimental setup

To evaluate the performance of the GP and WT-based image denoising approach, experiments have been conducted on real-world noisy images. All methods were implemented under identical conditions. Quantitative and qualitative metrics were employed to analyze both noise reduction capability and detail preservation. The denoising process is implemented in-house using Python 3.12, leveraging the OpenCV library (for image processing), the NumPy library (for numerical operations), PyWavelets, and scikit-image. All experiments were conducted on an AMD Ryzen 7 processor with Radeon graphics, 2900 MHz, 8-core (16 GB RAM).

To validate the efficiency of GP and WT methods, datasets that capture the characteristics of real-world images are utilized. We have used medical images (MRI and X-Ray), non-medical images, and SIDD datasets.

### MRI dataset

The Brain Tumor MRI Dataset, created by Masoud Nickparvar in 2021, is a collection of Magnetic Resonance Imaging (MRI) scans labeled for the presence or absence of brain tumors, mostly in JPEG/PNG format. This dataset is a combination of three different datasets, namely Fighshare, SARTAJ, and Br35H, and contains 7023 images. This dataset comprises 7023 human brain MRI images of varying sizes, aggregated from three different sets, namely SARTAJ, Fighshare, and Br35H. These are further classified into 4 classes: glioma, meningioma, no tumor, and pituitary^65^.

### X-ray dataset

This dataset, created by Paul Moony in 2018, comprises Chest X-ray images (anterior–posterior) that have been selected from retrospective cohorts of pediatric patients. There are 5856 X-Ray images (JPEG) and 2 categories (Pneumonia/Normal). 3883 images are characterized as depicting pneumonia (2538 bacterial and 1345 viral) and 1349 normal. In the test folder, 234 normal images and 390 pneumonia images (242 bacterial and 148 viral) from 624 patients^66,67^. No synthetic noise has been added; noise levels depend on the X-ray machine, patient movement, and exposure conditions.

### Non-medical images dataset

This dataset, created by Dibakar Sil in 2018, comprises natural images of 9 distinct categories. The images are corrupted by nine distinct types of noise, including additive Gaussian noise, lognormal noise, uniform noise, exponential noise, Poisson noise, salt and Pepper noise, Rayleigh noise, Speckle noise, and Erlang noise^68^.

### Smartphone image denoising dataset (SIDD)

The SIDD dataset, created by Rajat Gupta in 2020, comprises 160 pairs of noisy/ground-truth images taken under five different lightning conditions, including Google Pixel, iPhone 7, Samsung Galaxy Nexus 6, Motorola Nexus 6, and LG G4. The authors have provided a real noisy images dataset with high-quality ground truth. The noise is complex and signal-dependent^56^_._

## Results and discussion

This section analyzes the performance of the GP image denoising method and compares it with wavelet methods Coif4, Haar, dv4, and Sym. Both GP and WT are multiresolution approaches. Performance is evaluated using eight standard metrics: PSNR, SSIM, MSE, RMSE, MAE, VIF, FOM, and CC. The performance of GP is compared with the findings of WT methods, BM3D, and learning-based approaches, including DnCNN, RIDNet, and Noise2Void. The datasets have images with varying spatial resolutions. Denoising is performed at the original resolution, and the outputs are resized back to their original dimensions. For a comprehensive evaluation, the results are presented and discussed dataset-wise.

### X–ray dataset

For the X-ray dataset, GP exhibits superior performance compared to WT. Table 4 presents the quantitative results for image denoising using GP and WT methods. It achieves a higher PSNR of 36.8023 dB, indicating minimal distortion and effective preservation of intensity levels, which is highly important for retaining diagnostically important information in X-ray images. We used a five-layer GP to decompose the image at various resolutions. Bilateral, median, and Gaussian filters have been applied at different levels to suppress noise across multiple scales. The BF applied at the finest GP level is sensitive to edges and textures, removing high-frequency noise. Intermediate levels utilize median filters to remove salt-and-pepper noise without blurring anatomical structures such as bones and tissues, while Gaussian filters at higher levels eliminate low-frequency and spatially uniform background noise.

**Table 4:** Quantitative results of X-ray image denoising using soft thresholding, in terms of PSNR, SSIM, MSE, RMSE, VIF, FOM, MAE, and processing time metrics.

Method	PSNR (dB)	SSIM	MSE	RMSE	VIF	FOM	MAE	Time (sec)
Gaussian-Pyramid	36.802396	0.9428098	15.64632606	3.8416323	0.771763	0.992644	3.0442557	0.0046311
Wavelet-coif4	25.737732	0.790638	178.1433303	12.892460	0.710824	0.992018	12.9735588	0.1743912
Wavelet-db4	25.640081	0.788820	184.5384376	12.484285	0.710745	0.991923	13.1610439	0.1062235
Wavelet-Haar	26.6164154	0.788600	143.4125618	11.854359	0.714757	0.993376	11.6889089	0.094263
Wavelet-sym4	26.109799	0.783041	162.6687426	12.322558	0.712183	0.992352	12.4141359	0.1015763

So, multilevel filtering effectively addresses each type of noise. Additionally, the SSIM value of 0.9428 is high. SSIM assesses luminance, contrast, and structural correlations, making it sensitive to edge clarity and the spatial arrangement of pixels. This demonstrates the method’s ability to preserve details. Furthermore, the MSE value of 15.646 is lower than that of the wavelet method, indicating minimal pixel-level errors. The reduction in MSE alongside enhanced perceptual quality substantiates the robustness of the GP method for medical image denoising. Its localized, level-specific approach also offers a balance between accuracy and speed. Wavelet methods Coif4, db4, Haar, and Sym4 show PSNR values of 25.7377, 25.6400, 26.616, and 26.109 dB, respectively. Wavelet methods tend to introduce artifacts that impair pixel accuracy. Although WT effectively addresses high and low-frequency components, it often struggles to preserve edges. This leads to blurring of anatomical boundaries, reducing SSIM to 0.788. Higher values of VIF (0.7717) and FOM (0.992) also demonstrate the effectiveness of GP in preserving perceptual qualities well. Wavelet-based denoising employs predefined basic functions such as Haar and Daubechies, which may not be optimal for real-world images. A higher MSE between the original and denoised images indicates the presence of artifacts. Unlike GP, wavelet methods do not utilize a scale-specific filter. The computational efficiency of 0.0046 for GP is also notable, demonstrating that this method has a higher processing speed compared to all four WT methods. Table 5 shows the t-test results and the Wilcoxon test, which further confirm that GP performs significantly better than WT methods, with a t-value exceeding 19, a *p*-value of less than 0.0001, and a W value of 0.

**Table 5:** t-test and Wilcoxon test analysis for X-ray Dataset.

Filters	Paired t-test (t, *p*)	Wilcoxon test (W, *p*)	Significance
Gaussian Pyramid vs Wavelet-Coif4	t = 21.873, *p* = 0.0000	W = 0.0, *p* = 0.001953125	Significant
Gaussian Pyramid vs Wavelet-db4	t = 21.420, *p* = 0.0000	W = 0.0, *p* = 0.001953125	Significant
Gaussian Pyramid vs Wavelet-Haar	t = 19.873, *p* = 0.0000	W = 0.0, *p* = 0.001953125	Significant
Gaussian Pyramid vs Wavelet-sym4	t = 19.425, *p* = 0.0000	W = 0.0, *p* = 0.001953125	Significant

**Fig. 8:**
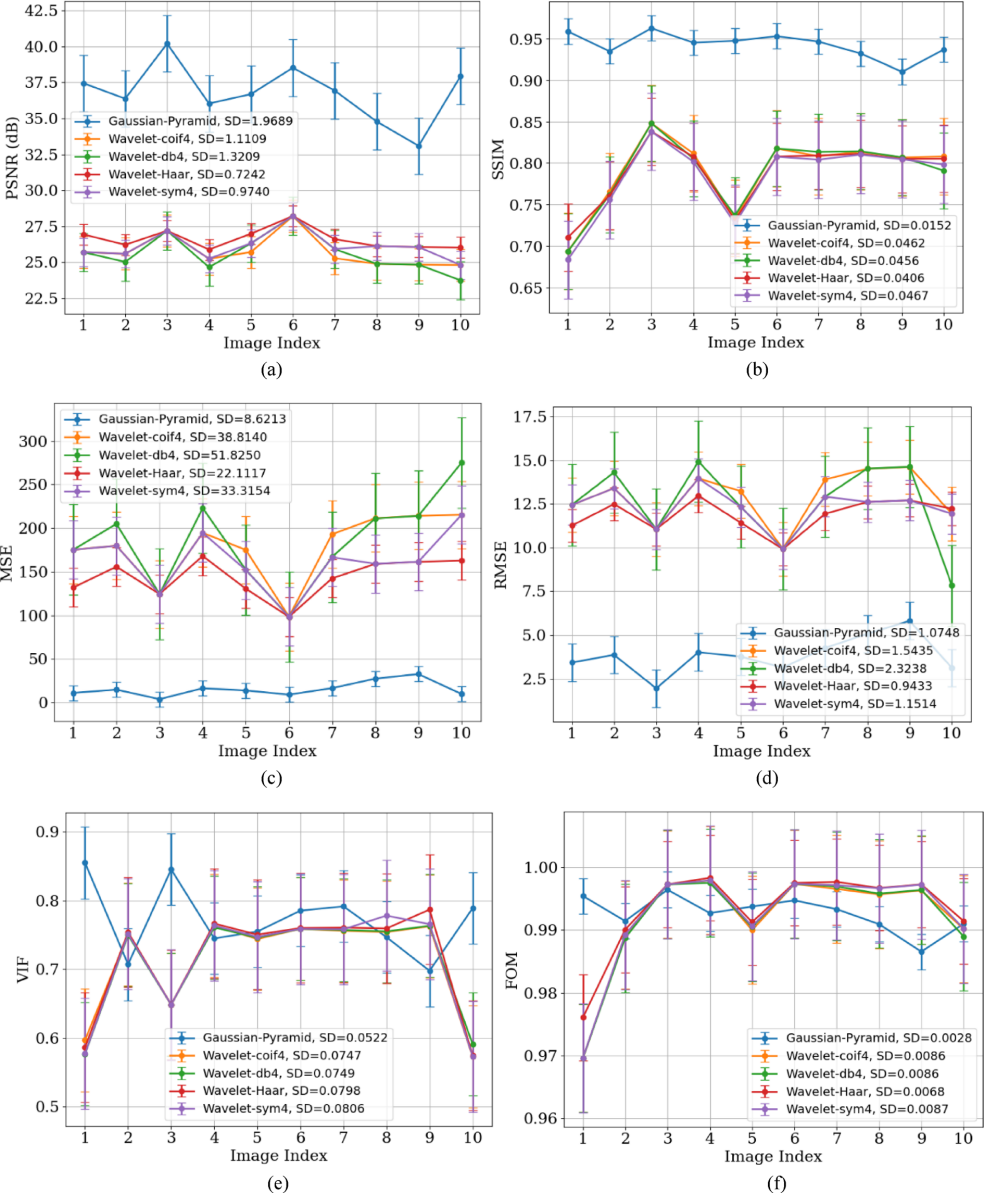

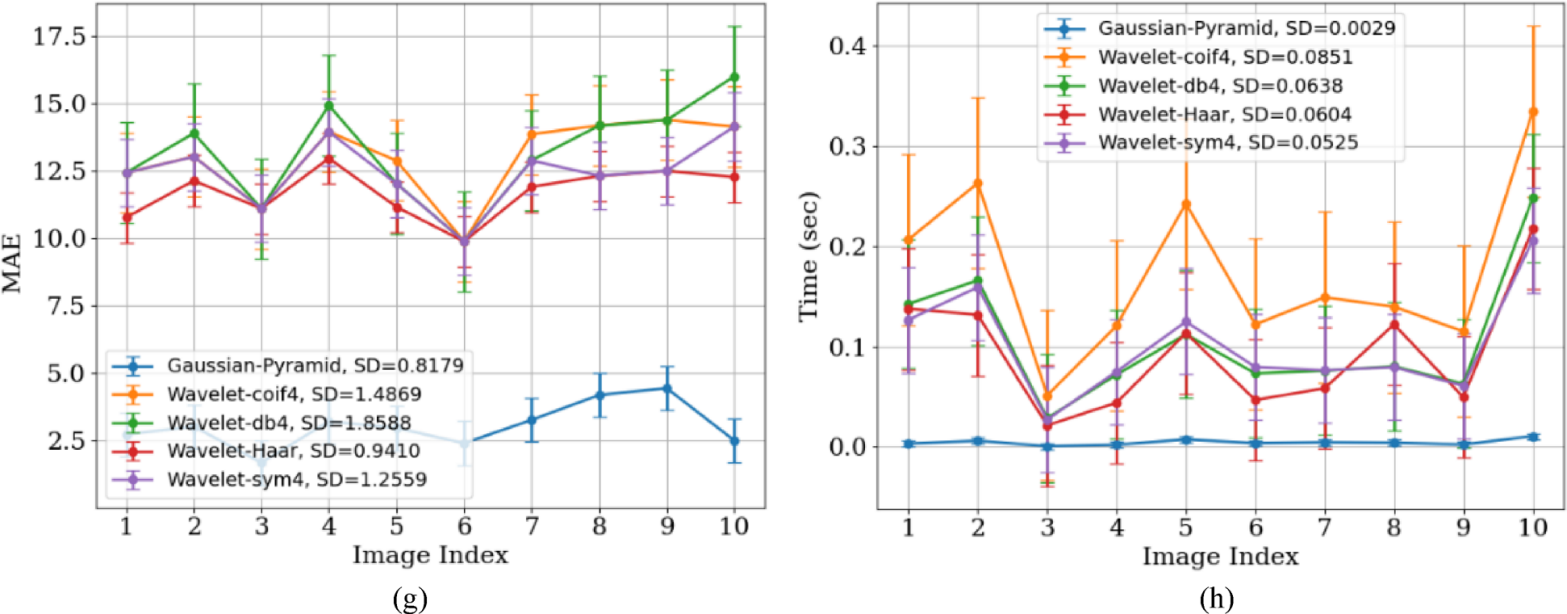
Performance analysis of GP and Wavelet (Coif4, Haar, db4, and Sym) methods using (**a**) PSNR, (**b**) SSIM, (**c**) MSE, (**d**) RMSE, (**e**) VIF, (**f**) FOM, (**g**) MAE, and (**h**) processing time metrics for the X-Ray dataset.

Figure 8 shows the variation of all parametric values across different X-ray images. These images, captured under different conditions (such as sensor noise, exposure, and varying radiation levels), contain complex, multi-level noise. Images with large uniform regions (like bone scans) tend to have relatively higher PSNR. PSNR also varies depending on the image content.

The lower SD value of 1.11 for WT indicates that these methods aggressively smooth the image intensities and also eliminate fine details. GP, with high PSNR and SSIM, preserves the structural details of the image, resulting in a higher SD.

**Fig. 9:**
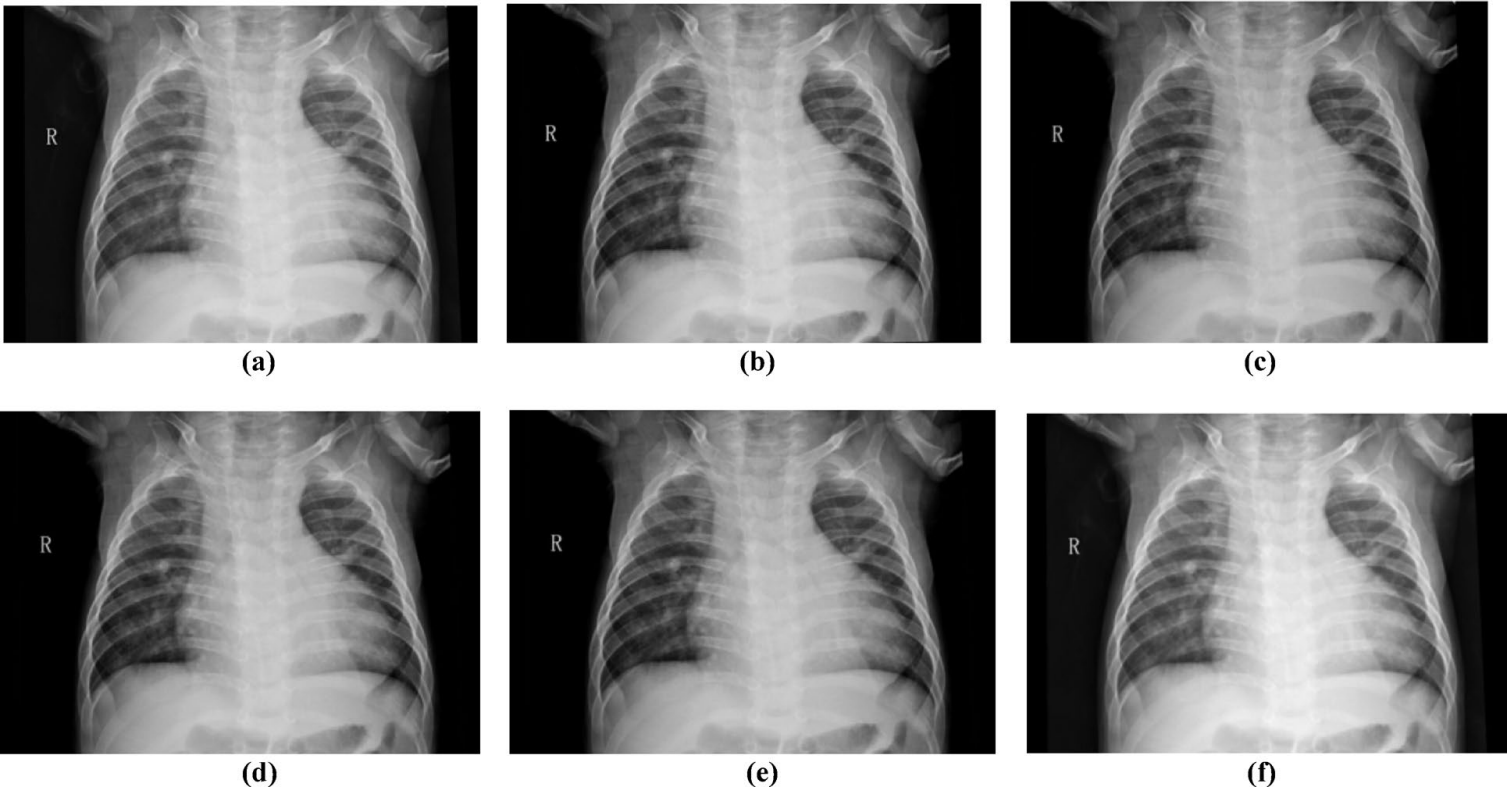
Visual comparison of X-ray images under different denoising methods. (**a**) Original noisy image, denoised image using(**b**) Gaussian Pyramid, (**c**) wavelet-Haar, (**d**) wavelet-coif4, (**e**) wavelet-db4, and (**f**) wavelet-sym4.

Figure 9 shows a visual comparison of the original images and denoised images by the GP and WT methods, which further supports the superiority of the GP method, as Fig. 9b presents better visual performance relative to the WT methods.

The lower SD value of 1.11 for WT indicates that these methods aggressively smooth the image intensities and also eliminate fine details. GP, with high PSNR and SSIM, preserves the structural details of the image, resulting in a higher SD.

### MRI dataset

**Fig. 10:**
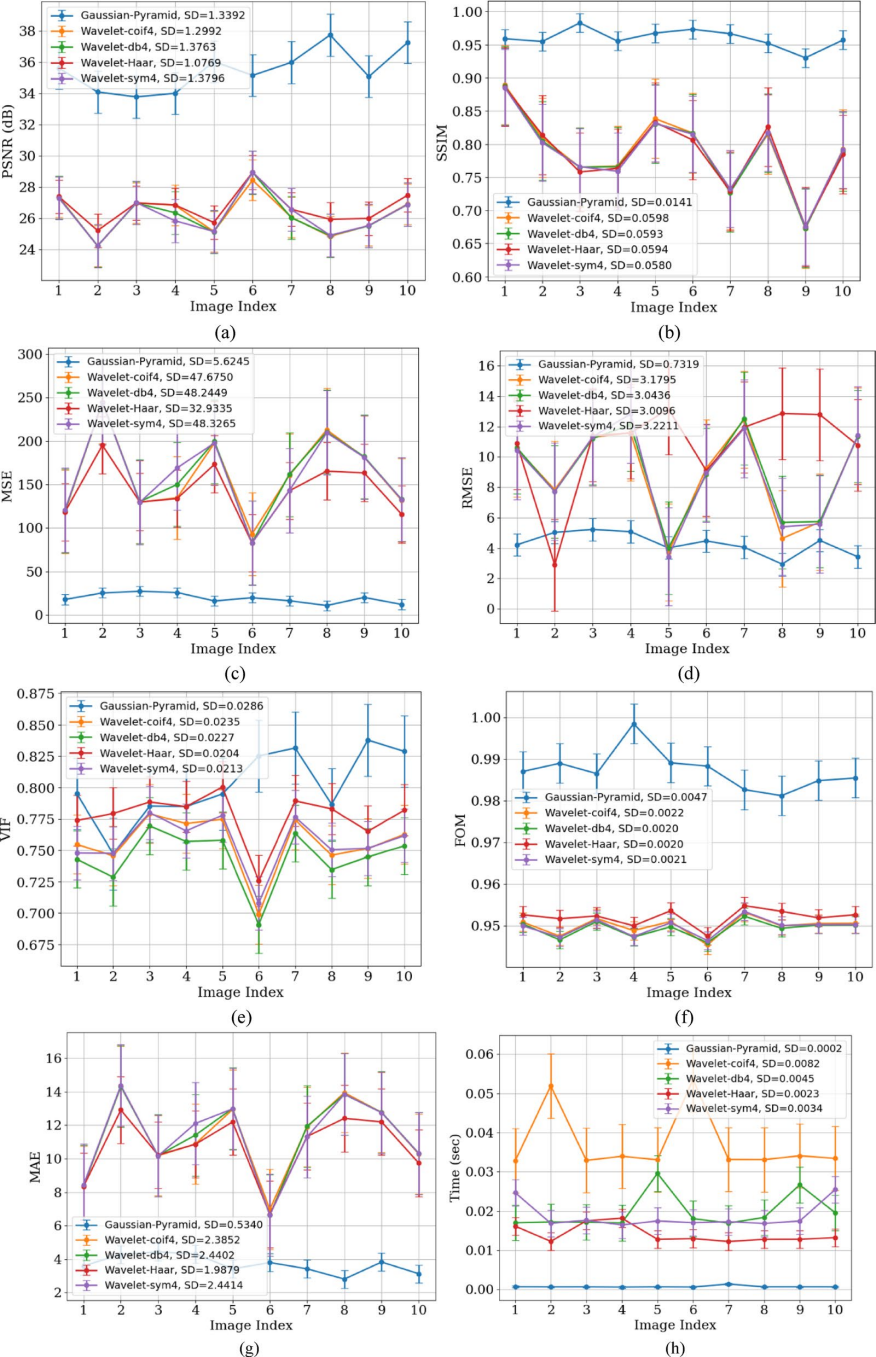
Performance analysis of GP and Wavelet (Coif4, Haar, db4, and Sym) using (**a**) PSNR, (**b**) SSIM, (**c**) MSE, (**d**) RMSE (**e**) VIF, (**f**) FOM, (**g**) MAE, and (**h**) processing time metrics for the MRI dataset.

**Fig. 11:**
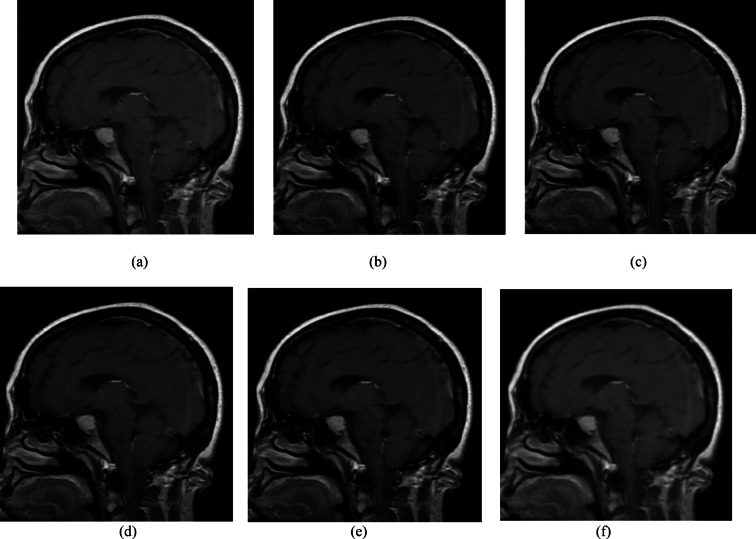
Visual comparison of MRI images under different denoising methods. (**a**) Original noisy image, denoised image using(**b**) Gaussian Pyramid, (**c**) wavelet-Haar, (**d**) wavelet-coif4, (**e**) wavelet-db4, and (**f**) wavelet-sym4.

In the MRI dataset, the GP method again yielded higher PSNR and SSIM values, indicating its robustness and effectiveness in noise suppression. Table 6 shows that the GP method exhibits a PSNR of 35.2776 dB and an SSIM of 0.9601 for the MRI dataset. BF, MF, and GF have been applied at different levels to reduce noise at various scales. As discussed for X-ray images analysis, the high PSNR is due to the multi-scale denoising performed in GP. In contrast, wavelet methods Coif4, db4, Haar, and Sym4 show PSNR values of 26.1197, 26.1154, 26.6431, and 26.1282 dB, respectively, indicating limited performance. The SSIM of 0.96013 indicates strong preservation of anatomical structures and higher values of VIF and FOM, demonstrating improved perceptual quality of GP compared to WT methods. Conversely, wavelet methods produced SSIM values ranging from 0.7763 to 0.7785, suggesting lower perceptual quality. With an MSE of 19.96, GP outperforms the others, while wavelet methods show higher MSE values, indicating the presence of noise artifacts. The low computational time of 0.00728 s provided by the GP denoising method confirms that it is highly effective for denoising MRI images. Figure 10 shows the performance of denoising methods across 8 different parameters. Figure 10a presents the lower SD value of 1.29 for WT, indicating that these methods aggressively smooth the image intensities and also suppress the fine details. Figure 11 shows the visual comparison of original images and denoised images using GP and WT variants. Figure 11b also favours that GP outperforms the wavelet methods with better visual performance demonstrated by the GP methods.

**Table 6:** Quantitative results of MRI image denoising using soft thresholding, in terms of PSNR, SSIM, MSE, RMSE, VIF, FOM, MAE, and processing time metrics.

Method	PSNR (dB)	SSIM	MSE	RMSE	VIF	FOM	MAE	Time (sec)
Gaussian-Pyramid	35.27762	0.96013	19.96940311	4.392524	0.798748	0.987508	3.767851	0.00728
Wavelet-coif4	26.11971	0.778557	165.2687697	8.653027	0.756028	0.949889	11.57544	0.037708
Wavelet-db4	26.11547	0.776344	166.0353806	8.798707	0.744509	0.949199	11.59846	0.020067
Wavelet-Haar	26.64319	0.776458	144.7506981	10.721	0.777606	0.952023	10.94744	0.013865
Wavelet- sym4	26.12824	0.7765	165.6444639	8.722778	0.757699	0.949719	11.60723	0.018065

Table 7 presents the paired t-test results and Wilcoxon test analysis, which also indicate that GP significantly outperforms WT-based methods, with a t-value exceeding 14, a *p*-value less than 0.0001, and W equals zero for 10 images. WT achieves lower SD by aggressively smoothing image intensities; however, it tends to over-smooth intensity variations and can also remove fine details and edges. On the other hand, GP retains multilevel structures. This demonstrates that GP performs more consistently than WT, which has an SD of 1.33 across the dataset.

**Table 7:** t-test and Wilcoxon test analysis for MRI Dataset.

Filters	Paired t-test (t, *p*)	Wilcoxon test (W,*p*)	Significance
Gaussian Pyramid vs Wavelet-Coif4	t = 14.613, *p* = 0.0000	W = 0.0, *p* = 0.001953125	Significant
Gaussian Pyramid vs Wavelet-db4	t = 14.483, *p* = 0.0000	W = 0.0, *p* = 0.001953125	Significant
Gaussian Pyramid vs Wavelet-Haar	t = 16.149, *p* = 0.0000	W = 0.0, *p* = 0.001953125	Significant
Gaussian Pyramid vs Wavelet-sym4	t = 14.883, *p* = 0.0000	W = 0.0, *p* = 0.001953125	Significant

The lower SSIM of X-ray images (0.9428) compared to MRI images (0.0.96013) using the GP method is because MRI images have higher soft tissue contrast and a broader grayscale dynamic range than X-ray images. Additionally, X-ray images, especially chest X-rays, contain many sharp anatomical edges (e.g., ribs and bone margins), which make them more prone to distortion or blurring during denoising. In contrast, MRI images generally have smooth anatomical transitions, making them less vulnerable to structural degradation and resulting in higher SSIM values. Noise suppression and signal retention result in higher PSNR in some images. Images with less contrast between anatomical structures tend to have lower PSNR. In smooth regions (e.g., white matter in MRI), even small noise can decrease PSNR, while in textured areas (e.g., grey matter), the same level of smoothing may be statistically beneficial but more visually or regionally significant. Therefore, PSNR may vary across images.

Figures 8a and 10a show that GP results in lower SD on MRI because these images contain large, homogeneous regions like soft tissues, and GP effectively removes random fluctuations in these areas, which reduces the residual intensity spread (SD). In contrast, X-rays have high-frequency structures (ribs and edges) and Poisson-like noise that GP intentionally preserves, leading to higher SD.

### Non-medical images dataset

In this dataset, GP outperformed WT by achieving higher PSNR and SSIM scores, demonstrating its ability to reconstruct images accurately and reliably. Table 8 shows that the GP method achieves a significantly higher PSNR of 25.04 dB as compared to WT methods, which have PSNR values ranging from 21.89 to 22.062 dB. This difference demonstrates the method’s ability to denoise images more effectively. SSIM values of GP (0.61133) and WT (0.60138) are comparable, as it is less sensitive to fine pixel-wise variation, while VIF and FOM highlight GP’s strength in retaining fine details. In medical images, where anatomical fidelity is responsive to gray-level variations, GP achieves clear improvement in SSIM. PSNR shows a notable difference between the two methods because it is highly sensitive to pixel-wise errors. Lower PSNR and SSIM values are observed for non-medical images due to their complex textures and high-frequency details, making denoising more challenging compared to medical images, which tend to have more uniform structures with fewer textures.

**Table 8:** Quantitative results of Non-medical Image denoising using soft thresholding in terms of PSNR, SSIM, MSE, RMSE, VIF, FOM, MAE, and processing time.

Method	PSNR (dB)	SSIM	MSE	RMSE	VIF	FOM	MAE	Time (sec)
Gaussian-Pyramid	25.0428	0.613304	242.7374	8.332471	0.509946	0.913019	12.1395	0.000606
Wavelet-coif4	21.89536	0.60138	438.1014	9.812835	0.457535	0.89582	16.33636	0.019436
Wavelet-db4	21.91733	0.608631	435.9906	9.79371	0.457324	0.895776	16.27617	0.010163
Wavelet-Haar	22.062	0.604246	422.319	9.717808	0.458314	0.89618	15.89167	0.008744
Wavelet-sym4	21.91765	0.608733	435.8237	9.797592	0.457542	0.895827	16.27397	0.01076

**Fig. 12:**
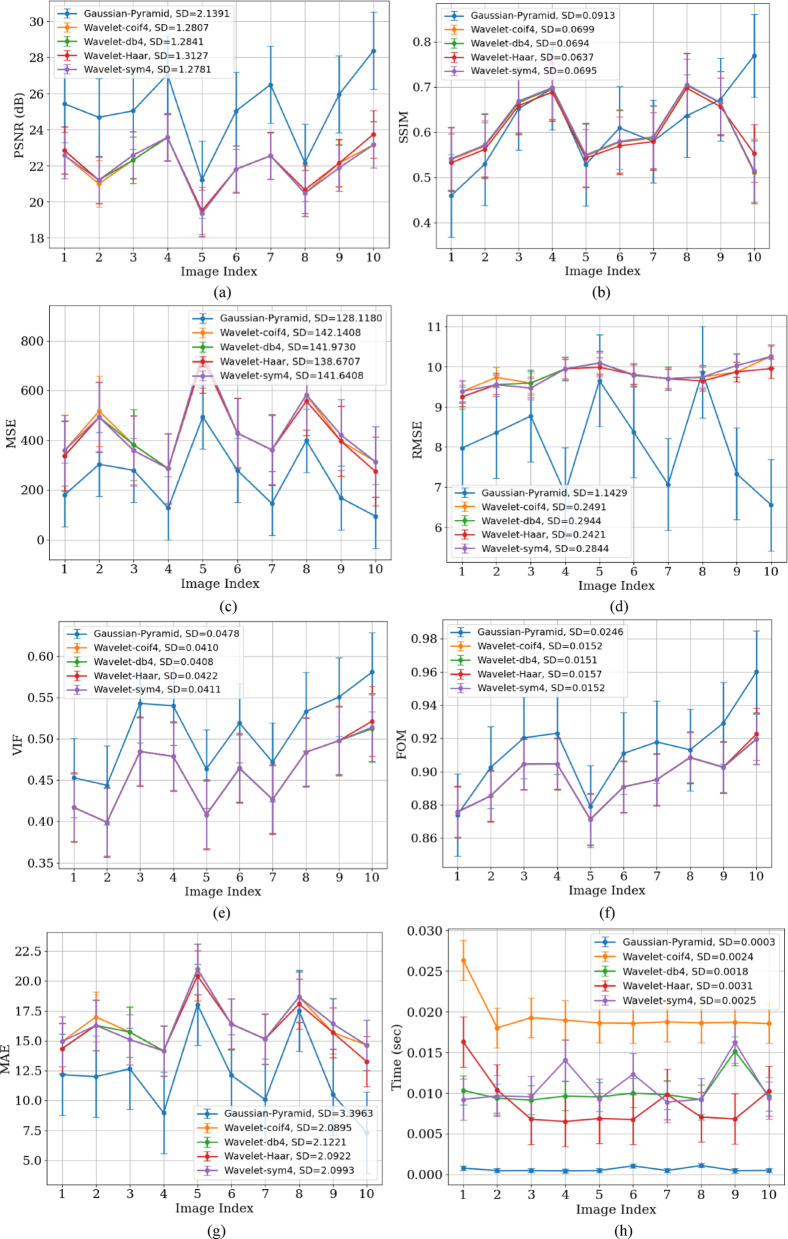
Performance analysis of GP and Wavelet (Coif4, Haar, db4, and Sym) using (**a**) PSNR, (**b**) SSIM, (**c**) MSE, (**d**) RMSE (**e**) VIF, (**f**) FOM, (**g**) MAE, and (**h**) processing time metrics for the NMI dataset.

Figure 12 exhibits the parameter values for different images. Although wavelet transforms (Haar, db4, and sym4) are well-suited for multi-resolution analysis, these appear less effective for real-world image denoising when compared to the GP method. Variations in PSNR values across different non-medical images occur because these images vary significantly, such as in terms of skies, walls, and water. Images with smooth regions tend to have higher PSNR, while fine textures and edges are more susceptible to noise suppression, resulting in lower PSNR values. Figure 12(a) shows that for non-medical images, the SD variation in GP and WT is small (1.28), as compared to medical images (2.13), because their complex and diverse intensity patterns overshadow the impact of variance reduction. Conversely, in medical images with large homogeneous regions, denoisers have a stronger effect on pixel variance, resulting in a noticeable gap between SD values for these two methods.

**Fig. 13:**
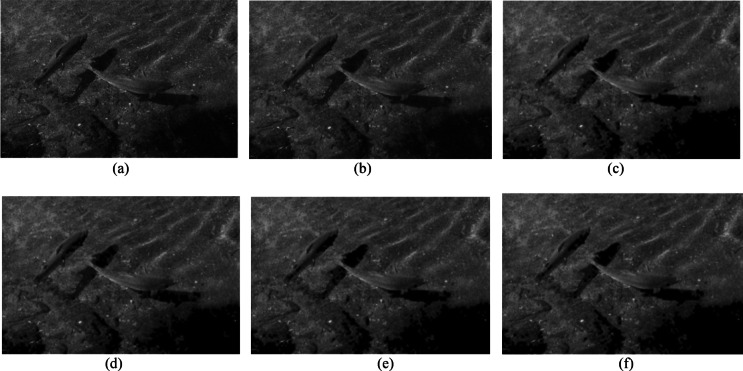
Visual comparison of NMI images under different denoising methods. (**a**) Original noisy image, denoised image using(**b**) Gaussian Pyramid, (**c**) wavelet-Haar, (**d**) wavelet-coif4, (**e**) wavelet-db4, and (**f**) wavelet-sym4.

Table 9 displays the t-test and Wilcoxon test values for the NMI dataset, which also support the finding that GP performs significantly better than WT methods, with a t-value exceeding 11, a *p*-value of less than 0.0001, and a W score of zero. Figure 13 shows the visual comparison denoised image. Visual comparison further supports the findings. Figure 13(b) shows that the GP-based method generated denoised images with finer structure and fewer artifacts compared to WT.

**Table 9:** t-test and Wilcoxon test analysis for NMI Dataset.

Filters	Paired t-test (t, *p*)	Wilcoxon test (W, *p*)	Significance
Gaussian Pyramid vs Wavelet-Coif4	t = 11.689, *p* = 0.0000	W = 0.0, *p* = 0.001953125	Significant
Gaussian Pyramid vs Wavelet-db4	t = 11.118, *p* = 0.0000	W = 0.0, *p* = 0.001953125	Significant
Gaussian Pyramid vs Wavelet-Haar	t = 11.464, *p* = 0.0000	W = 0.0, *p* = 0.001953125	Significant
Gaussian Pyramid vs Wavelet-sym4	t = 11.059, *p* = 0.0000	W = 0.0, *p* = 0.001953125	Significant

### SIDD dataset

**Fig. 14:**
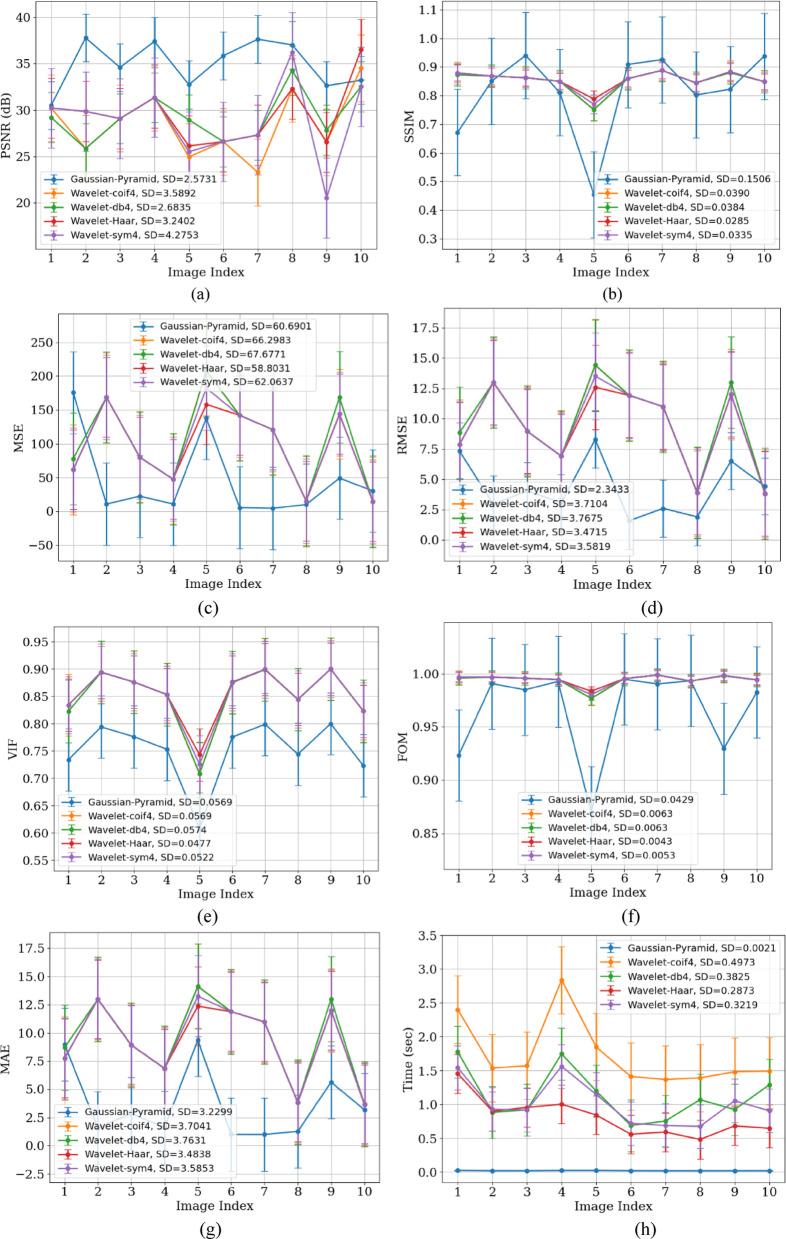
Performance analysis of GP and Wavelet (Coif4, Haar, db4, and Sym) using (**a**) PSNR, (**b**) SSIM, (**c**) MSE, (**d**) RMSE (**e**) VIF, (**f**) FOM, (**g**) MAE, and (**h**) processing time metrics for the SIDD dataset.

In this dataset, GP maintained its consistency in reconstruction capability with a higher PSNR. Table 10 shows that GP achieves higher pixel-level accuracy, reflected in higher PSNR (34.9333 dB) and lower MSE and RMSE, while WT exhibits higher SSIM and VIF for the SIDD dataset. Therefore, for natural images, GP is more effective at reducing reconstruction error and computational complexity, whereas WT emphasizes the preservation of global and perceptual content. The high variations in PSNR across the SIDD dataset images are due to heterogeneous noise levels, diverse scene content, and preprocessing differences. Figure 14 exhibits the performance analysis of GP and WT methods using PSNR, SSIM, MSE, RMSE, VIF, FOM, MAE, and computational complexity.

**Table 10:** Quantitative results of SIDD Image denoising using soft thresholding, in terms of PSNR, SSIM, MSE, RMSE, VIF, FOM, MAE, and processing time.

Method	PSNR (dB)	SSIM	MSE	RMSE	VIF	FOM	MAE	Time (sec)
Gaussian-Pyramid	34.93335	0.812642	45.77423	4.269176	0.750642	0.96519	3.651002	0.018795
Wavelet-coif4	29.47516	0.853745	100.2742	9.37451	0.850642	0.99406	9.306391	1.732171
Wavelet-db4	29.3042	0.852817	104.4029	9.571381	0.84939	0.993929	9.501932	1.122242
Wavelet-Haar	29.59382	0.857456	95.31909	9.190914	0.854258	0.994739	9.129476	0.809155
Wavelet-sym4	29.53238	0.855697	97.7224	9.283064	0.852461	0.994415	9.218088	1.011399

**Fig. 15:**
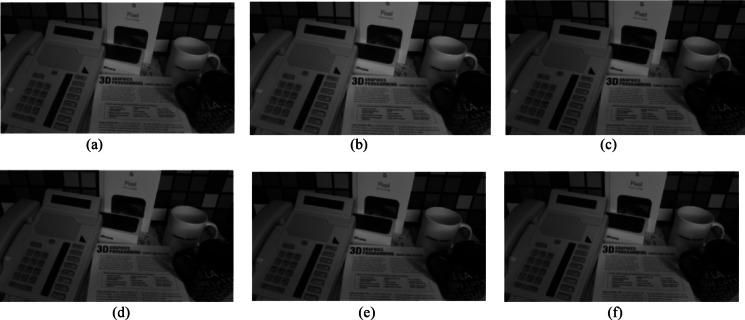
Visual comparison of SIDD images under different denoising methods. (**a**) Original noisy image,denoised image using(**b**) Gaussian Pyramid, (**c**) wavelet-Haar, (**d**) wavelet-coif4, (**e**) wavelet-db4, and (**f**) wavelet-sym4.

To validate the results, statistical analysis is also performed using t-tests and the Wilcoxon signed-rank test, which also confirmed the superiority of GP over wavelet-based methods. Table 11 shows paired t-tests with a t-value of 4.131 and a *p*-value of 0.0026, along with a Wilcoxon test showing a w-score of 2.0 and a *p*-value of 0.00585, supporting the robustness of GP compared to WT. In Fig. 14a lower SD (2.57) of GP demonstrates that it is highly sensitive to scene variability, achieving higher accuracy in smooth areas but large fluctuations in texture-rich regions. Figure 15 presents the visual comparison for this dataset. GP produced cleaner images with preserved information and minimal artifacts.

**Table 11:** t-test and Wilcoxon test analysis for SIDD Dataset.

Filters	Paired t-test (t, *p*)	Wilcoxon test (W, *p*)	Significance
Gaussian Pyramid vs Wavelet-Coif4	t = 4.289, *p* = 0.0020	W = 2.0, *p* = 0.005859375	Significant
Gaussian Pyramid vs Wavelet-db4	t = 4.670, *p* = 0.0012	W = 0.0, *p* = 0.001953125	Significant
Gaussian Pyramid vs Wavelet-Haar	t = 4.131, *p* = 0.0026	W = 2.0, *p* = 0.005859375	Significant
Gaussian Pyramid vs Wavelet-sym4	t = 4.508, *p* = 0.0015	W = 0.0, *p* = 0.001953125	Significant

Table 12 presents the effect of soft and hard thresholding used in wavelet methods. The improved PSNR (ranging from 0.5 to 1.0 dB) and SSIM (ranging from 0.18 to 0.20) using soft thresholding are attributable to its gradual shrinkage function applied to coefficients, whereas in hard thresholding, coefficients below a threshold are set to zero, directly resulting in discontinuities in the image. This improved reconstruction ability comes at the cost of increased computational time due to the additional processing required for coefficient adjustment. In contrast, high thresholding offers faster execution at the expense of compromised image quality, as it eliminates fine details and noise.

**Table 12:** Comparison of soft and hard thresholding for Wavelet Transform–based denoising.

Method	Soft Thresholding			Hard thresholding		
	**PSNR (dB)**	**SSIM**	**Time (sec)**	**PSNR (dB)**	**SSIM**	**Time (sec)**
Wavelet- coif4	26.2458334	0.78962	0.037219	25.748822	0.601735	0.032492
Wavelet-db4	26.2386299	0.787474	0.019762	25.554717	0.593137	0.015048
Wavelet-Haar	26.7183094	0.787426	0.014093	25.568394	0.593076	0.010545
Wavelet-sym4	26.2462602	0.787385	0.018727	25.604334	0.583709	0.01076

This work highlights the strength of a GP framework for real-world image denoising.

Table 13 presents a comparative analysis of GP and WT-based methods against the benchmarked learning-based denoising methods, including DnCNN, RIDNet, and Noise2Void. The results show that GP outperforms WT, producing higher PSNR and SSIM with less computational complexity. Results are competitive with these state-of-the-art learning-based methods. All of these require extensive training datasets, have high computational costs, and are more resource-intensive. In contrast, the GP method is lightweight, training-free, and adaptable across diverse datasets. For medical images, it achieves a PSNR of more than 35 dB with an SSIM of 0.96013, comparable to RIDNet (0.9526) and DnCNN (0.8635) and higher than Noise2Void (27.71), indicating that GP not only suppresses noise but also maintains structural integrity. This demonstrates that GP is an interpretable, efficient, and reliable alternative for real-world scenarios where both accuracy and interpretability are important.

**Table 13:** Comparison of Gaussian Pyramid, Wavelet Transform, BM3D, and DL–based denoising methods in terms of PSNR, SSIM, computational time, and the dataset used.

Dataset used	Method	PSNR (dB)	SSIM	Time (sec)
X-ray	Gaussian-Pyramid	36.802396	0.942809	0.0046311
	Wavelet-coif4	25.737732	0.790638	0.1743912
	Wavelet-db4	25.640081	0.788820	0.1062235
	Wavelet-Haar	26.6164154	0.788600	0.094263
	Wavelet-sym4	26.109799	0.783041	0.1015763
MRI	Gaussian-Pyramid	35.27762	0.96013	0.00728
	Wavelet-coif4	26.11971	0.778557	0.037708
	Wavelet-db4	26.11547	0.776344	0.020067
	Wavelet-Haar	26.64319	0.776458	0.013865
	Wavelet-sym4	26.12824	0.7765	0.018065
SIDD	GaussianPyramid	34.93335	0.812642	0.018795
	Wavelet-coif4	29.47516	0.853745	1.732171
	Wavelet-db4	29.3042	0.852817	1.122242
	Wavelet-Haar	29.59382	0.857456	0.809155
	Wavelet-sym4	29.53238	0.855697	1.011399
BSD68	DnCNN^38^	33.86	0.8635	0.060
Set12	BM3D^69^	33.52	0.925	2.274
DnD	RIDNet^40^	39.23	0.9526	0.2
BSD68	Noise2Void^39^	27.71	–	0.1

The GP method effectively reduces high-frequency noise while preserving edges and finer. It is also computationally efficient and free of iterative optimization or DNN inference, making it suitable for real-time applications such as medical diagnosis, satellite imaging, underwater imaging, video surveillance, and robotics vision, where expedited noise reduction is necessary to improve workflow. As efficiency is a crucial characteristic of filters and transforms, it can be implemented even on resource-constrained platforms, such as embedded clinical image consoles and embedded systems.

## Challenges and limitations

Despite the promising results of the GP-based image denoising technique in terms of PSNR, SSIM, MSE, and computational cost for X-ray and MRI images, several challenges remain that require further attention. The performance of the GP denoising method highly depends on the choice of filters at various pyramid levels. Suboptimal settings can either over-smooth diagnostically important details at finer levels or leave residual noise at coarser levels. Although GP is less complex than deep learning techniques, its multiscale processing and reconstruction still require iterative operations, such as downsampling, filtering, and upsampling, which increase processing time compared to simple spatial filters. The effectiveness of the GP method is influenced by the content of the image; for example, high-contrast or textured images may retain more noise, while homogeneous regions such as soft tissues in MRI or skies and lakes in non-medical images may benefit more. Medical images can also contain motion artifacts. More complex artifacts, such as Poisson noise from low-dose acquisition, streaks caused by motion, or coil-related intensity heterogeneity, are only partially reduced, indicating a need for hybrid noise models. Unlike deep learning methods, GP is manually parameterized, which limits its ability to generalize across heterogeneous datasets. Unlike deep learning methods, GP is manually parameterized, which limits its ability to generalize across heterogeneous datasets and learning methods. This limits its generalizability across heterogeneous datasets and learning methods.

## Future directions

While the GP method shows significant improvements over traditional techniques, researchers continue to seek further enhancements. Exploring hybrid networks that combine GP with deep learning frameworks, such as CNNs or GANs, is promising. Developing adaptive and context-aware methods that can manage non-uniform noise distributions is recommended. Domain-specific applications, including medical and underwater imaging, along with multiscale and multimodal approaches, complemented by standardized benchmarks, offer promising directions for better denoising performance. Several aspects of GP decomposition remain open for future research, which could enhance adaptability by replacing fixed filters with adaptive ones. VAEs can be integrated at various layers of the pyramid to boost flexibility in image denoising. Future studies should focus on optimizing deep learning models, especially for unsupervised learning and real-time processing in large-scale image tasks. CC is more suitable for GP, whereas the Kalman filter excels in denoising efficiency; thus, combining GP with the Kalman filter could enhance adaptability and perceptual quality across different imaging modalities.

The GP denoising framework has low complexity and is not based on training; hence, it is easy to implement for real-time processing on system-on-chips, such as digital camera or smartphone image processing pipelines. Its hierarchical multi-scale structure provides excellent noise suppression and fine structural detail preservation capability, resulting in good image quality under low-light and high-noise-level conditions. Further studies may investigate the direct incorporation of the GP approach into on-device imaging platforms to demonstrate practical usability.

## Data Availability

No datasets were generated or analysed during the current study.

## Code availability

The code developed and used in this study is developed by the authors. It is not publicly available as it is being further developed for ongoing research. Reasonable requests for access to the code can be considered by contacting the corresponding author.
